# Harmonic vowels and neural dynamics: MEG evidence for auditory resonance integration in singing

**DOI:** 10.3389/fnins.2025.1625403

**Published:** 2025-08-13

**Authors:** Wolfgang Saus, Annemarie Seither-Preisler, Peter Schneider

**Affiliations:** ^1^Section of Biomagnetism, Department of Neurology, University of Heidelberg Medical School, Heidelberg, Germany; ^2^School of Overtone Singing, Dürrwangen, Germany; ^3^Music Psychology and Brain Research Section, Department of Psychology, University of Graz, Graz, Austria; ^4^Division of Neuroradiology, University of Heidelberg Medical School, Heidelberg, Germany; ^5^Latvian Academy of Music, Riga, Latvia

**Keywords:** vowel resonance, auditory flexibility, theta oscillations, gamma oscillations, overtone perception, pitch perception mode

## Abstract

**Introduction:**

Auditory perception of sung syllables involves rapid shifts between speech-like interpretation and spectral awareness of resonance. Perceiving vocal tract resonances as pitch-like elements may be crucial for singers, linking this concept to pedagogical practice and underlying neural mechanisms. This study examines how vowel resonance becomes accessible to conscious processing and how such perceptual shifts are reflected in neural dynamics.

**Methods:**

Drawing on a novel acoustic-phonetic model of “harmonic vowels,” we presented sung syllables that varied systematically across six distinct conditions, ranging from speech-like utterances to overtone singing. Magnetoencephalographic (MEG) recordings from 17 participants revealed distinct modulations in cortical oscillatory activity.

**Results:**

Theta-band power (4–7 Hz) increased linearly with decreasing speech content and showed strong right-hemispheric lateralization (partial η² = 0.82), indicating a key role in the cortical representation of spectral content. Gamma-band power (30–60 Hz) declined moderately and was left-lateralized. These findings show that vowel resonance is perceptually accessible and subject to rapid auditory reorientation, reflecting neural flexibility that may underlie auditory plasticity in both trained and untrained listeners. Individual differences in pitch perception mode (fundamental vs. overtone-based), indicating a stable perceptual trait, were also systematically reflected in oscillatory patterns: overtone listeners exhibited higher theta power, lower gamma power, and stronger right-hemispheric lateralization in both bands.

**Discussion:**

Theta and gamma power were inversely correlated, suggesting complementary functions in detail-oriented spectral representations and global feature binding. The results offer novel implications for vocal pedagogy, auditory training, and sound-based therapeutic applications.

## Introduction

Singing is primarily shaped by vowels (Nair, 2021). The resonance characteristics of both sung and spoken vowels significantly influence vocal projection, brilliance, and stability (Seidner and Wendler, 2004; Sundberg, 2015; Titze et al., 2008; Titze, 2008). Vowels are characterized by the two lower vocal tract resonance frequencies—typically referred to as the first and second formants. This resonance pair shapes the amplitude distribution of the voice’s harmonics and thus determines the perceived vowel quality. Maximum energy transfer to a resonator occurs when the excitation frequency coincides with a resonance frequency (Neppert, 1999), a condition encountered in singing when vowel resonances align with harmonics of a fundamental frequency.

In classical singing (Sundberg, 2015), and in overtone singing in particular (Saus, 2004), resonance strategies take precedence over pronunciation, as they not only provide the necessary projection but also contribute to vocal stability or, in the case of overtone singing, create the desired sound effects. Understanding the alignment between harmonics and vocal tract resonances is therefore of crucial importance. Berton Coffin’s Chromatic Vowel Chart (Coffin, 1980, 1995) illustrated how vowel formants relate to pitch by mapping resonance frequencies onto musical notes. In his work, as well as in other representations of sung vowels (Bozeman, 2013; Bozeman and Spivey, 2021; Howell, 2016; Miller and Schutte, 1990), the authors draw on concepts from speech phonetics to model vowel perception. However, vowel descriptions from acoustic, auditory, and articulatory phonetics are too imprecise for accurate formant-to-partial matching. For example, formant frequencies for the vowel /u/ can differ by around a minor third within a single language and by up to a tritone between Swedish and German (Table 1).

**Table 1:** Formant frequencies for the vowel /u/ reported by exemplary authors, converted to pitches (c indicates the deviation in cents relative to equal-tempered tuning at 440 Hz).

Vowel /u/	fR1	fR2
Swedish, spoken (Fant et al., 1969)	290 Hz D4-21c	595 Hz D5 + 21c
Swedish, 4 s duration (Fant et al., 1969)	310 Hz E♭4-8c	730 Hz G♭5-23c
German (Rausch, 1972)	250 Hz B3 + 24c	668 Hz E5 + 24c
German (Neppert, 1999)	275 Hz D♭4-13c	850 Hz A♭5 + 39c

In contrast, the Singing Phonetics Diagram (Saus, 2017a) proposed here emphasizes the perception of acoustically measurable vowel resonance pitches, independently of traditional speech-based vowel categories. According to the first author’s experience as a singing pedagogue, only a small percentage of singers—estimated at around 5%—spontaneously perceive vowel resonances as distinct pitches; most singers are unaware that this type of perception is even possible. Historically, these “vowel tones” were described by some authors in the 19th century (Willis, 1829; von Helmholtz, 1857, 1859, 1896). Zacher (1989) suggested that the composer Johannes Brahms took vowel tones into account in the harmony of the piano accompaniment in the form of “composed formants” in the last of his *Vier ernste Gesänge*. However, this concept has not been adopted by modern vocal pedagogy, presumably because few people develop this type of pitch perception without targeted training. Despite this, it remains unclear whether singers can learn to consciously perceive resonance frequencies as pitch-like elements—and how such perceptual abilities might be reflected neurally.

This raises the first key question: Can singers learn to perceive resonance frequencies as distinct pitch elements? While uncommon, this ability would be crucial for effectively utilizing the Singing Phonetics Diagram, which offers a new perspective for understanding singing vowels. If resonance frequencies are experienced as pitch-like percepts, singers could intentionally modulate their vocal tract to optimize vowel resonances. By “optimizing vowel resonances,” we refer to the intentional adjustment of vocal tract shape to enhance or suppress specific resonance frequencies in the vocal tract by motor-auditory control, thereby influencing the acoustic quality, tonal color, projection and presence of the sung voice. To date, traditional voice pedagogy has largely overlooked this aspect, focusing instead on subjective vowel quality rather than the objective acoustic structure of vocal resonance.

To bridge this gap, the first author developed a perceptual training test designed to help singers recognize vowel resonances as distinct pitches. This structured auditory learning experience, termed Harmonious Awareness Training (HAT), systematically isolates vowel resonances and aligns them with musical intervals. Preliminary observations from over a decade of teaching suggest that this perceptual skill can be developed relatively quickly, pointing to a latent but trainable auditory processing capacity. This led to two further key questions: Which neural mechanisms underlie this perceptual transformation? And does training not only refine an existing ability, but also induce measurable neurophysiological changes?

Recent neuroimaging evidence suggests that harmonicity and speech-like acoustic features such as resolved harmonics and formant structure are processed in specialized auditory cortical circuits, particularly within anterior and lateral regions of the superior temporal gyrus (Albouy et al., 2024). These mechanisms support the perceptual extraction of pitch, vowel identity, and voice quality, even in the absence of lexical meaning. Thus, our stimuli—though semantically meaningless—likely engage similar neural systems involved in the early stages of speech and timbre perception.

Moreover, we hypothesize that the perception of vocal tract resonances is not driven solely by bottom-up acoustic features, but also depends on higher-order, top-down processes that classify sounds as either speech-like or music-like. This top-down categorization is crucial because identical or similar acoustic cues may be processed differently depending on whether listeners perceive them as part of speech or song, which in turn may influence hemispheric engagement and oscillatory dynamics (Poeppel, 2003; Poeppel and Assaneo, 2020; Zatorre and Gandour, 2008). Throughout the present work, we distinguish between two principal types of acoustic information relevant for auditory perception and production. On the one hand, pitch-based cues refer to the fundamental frequency (f_0_) or its virtual pitch derived from harmonic relationships, forming the basis for harmonic and melodic intervals. On the other hand, resonance-based cues encompass vocal tract resonances, which can be experienced as distinct spectral pitches or as global spectral qualities, giving rise to vocal timbre. Differentiating how brain activity relates to these two perceptual qualities is essential for understanding the auditory mechanisms underlying both vocal perception and production in speech and singing. The speech–song continuum is therefore highly relevant for our research questions, as we aim to explore how the brain differentiates pitch-based and resonance-based cues along this perceptual axis. This approach may help explain why speech perception is often associated with increased gamma-band activity linked to fine-grained temporal processing, while music and spectral listening may involve stronger theta-band oscillations (Poeppel and Assaneo, 2020).

Importantly, the present study focuses on the perception of externally presented vocal sounds rather than self-initiated vocalizations. Several studies have demonstrated distinct neural signatures for self-generated speech, such as N1 suppression and enhanced N2b/P3a responses to deviant self-initiated vowels, reflecting forward modelling and the salience of prediction errors (Knolle et al., 2013, 2019). This concept is closely linked to audio-motor feedback and feedforward loops described in musical performance, where auditory perception and motor production are dynamically integrated to fine-tune vocal output (Altenmüller et al., 2006). Understanding these neural loops is crucial, as they provide the physiological basis for translating perceptual awareness of resonance cues into precise vocal control. Our approach examines how listeners neurally and perceptually differentiate pitch-based and resonance-based cues when passively listening to vocal sounds. Notably, the underlying predictive mechanisms and error responses may differ between self-generated and externally generated auditory events.

While our primary focus is on singing, it is important to note that sung vocalizations often retain speech-like qualities, particularly with regard to articulation, intelligibility, and expressive timing. Therefore, understanding how vocal tract resonances are perceived and neurally represented is relevant not only for singing pedagogy but also for bridging the neural and perceptual mechanisms shared across the speech–song continuum. This broader framing allows for a more comprehensive investigation into how spectral and articulatory cues are processed within both musical and verbal vocalizations.

### Acoustic characteristics and individual differences in pitch perception

The perception of harmonic complex tones critically depends on the acoustic properties of the stimulus itself: factors such as the relative harmonic order, the number and density of harmonics (Preisler, 1993), and the spectral bandwidth of the harmonic complex (Seither-Preisler et al., 2003) can enhance or weaken the subjective salience of a missing fundamental frequency, directly influencing whether a listener perceives a virtual pitch. A central motivation of our study was to better understand how stimulus-driven influences, as systematically varied in the HAT paradigm, interact with individual differences in pitch perception. Specifically, some listeners consistently rely on the missing fundamental (F₀) as a pitch cue, while others base their pitch judgments on spectral (overtone) information. This distinction is not trivial, as numerous studies have shown that such perceptual preferences reflect distinct auditory strategies that are remarkably stable across individuals (Patel and Balaban, 2001; Renken et al., 2004; Schneider et al., 2005a, 2005b; Seither-Preisler et al., 2007; Ladd et al., 2013; Schneider et al., 2023). Importantly, musical expertise per se does not predict perceptual style. Rather, trained musicians may be specialized as well in fundamental as in spectral listening, depending on the played instrument (Schneider et al., 2005b). On the neuroanatomical level, Schneider et al. (2005a, 2005b) demonstrated that individuals with a preference for fundamental (f₀-based) perception tend to have larger left Heschl’s gyrus volumes and stronger early P1 responses in the left hemisphere, whereas individuals with a preference for spectral (overtone-based) listening show rightward dominance. These hemispheric asymmetries are exceptionally stable throughout childhood and adolescence (Schneider et al., 2023), pointing to a strong predispositional component. Our study therefore aimed to probe how stimulus characteristics and individual predispositions jointly shape auditory processing.

### Neural oscillations in pitch processing and auditory scene analysis

Importantly, differences between f_0_ and overtone listeners extend beyond brain structure to dynamic neural processing. Gamma-band oscillations have been associated with feature binding in pitch perception: Schulte et al. (2002) showed increased gamma power after training induced a perceptual switch to f_0_ perception, and Aksentijevic et al. (2013) found that gamma-range priming biases listeners toward virtual (f_0_) pitch.

Theta-band oscillations, in contrast, appear to support spectral (overtone) listening and auditory scene analysis. Tóth et al. (2016) and Kocsis (2017) both demonstrated that theta power increases significantly during conditions favouring the segregation of concurrent sound streams or multiple auditory objects, with the strongest effects observed over right-hemisphere electrodes. This is consistent with the right auditory cortex’s role in processing spectral detail and reinforces theta oscillations as a neural correlate of fine-grained auditory feature perception and auditory stream segregation.

These complementary roles of gamma and theta oscillations are conceptually consistent with the asymmetric sampling theory (AST) of Poeppel (2003), which proposes that the left and right auditory cortices preferentially process information in different temporal integration windows, potentially supporting distinct pitch perception strategies.

While substantial work has detailed the long-term stability of individual pitch perception styles and their structural and oscillatory correlates, it remains unclear how stable perceptual predispositions interact with stimulus-driven factors to shape immediate neural processing during pitch perception. Addressing this gap is crucial for understanding how individual auditory strategies and the acoustic properties of incoming sounds jointly influence cortical dynamics. Clarifying this interaction allows us to investigate how stable individual auditory strategies influence the immediate neural encoding of acoustic features, thus bridging predispositional differences and context-dependent auditory modulation.

### Theoretical background: model of vowel resonances

#### Harmonic vowels

**Figure 1:**
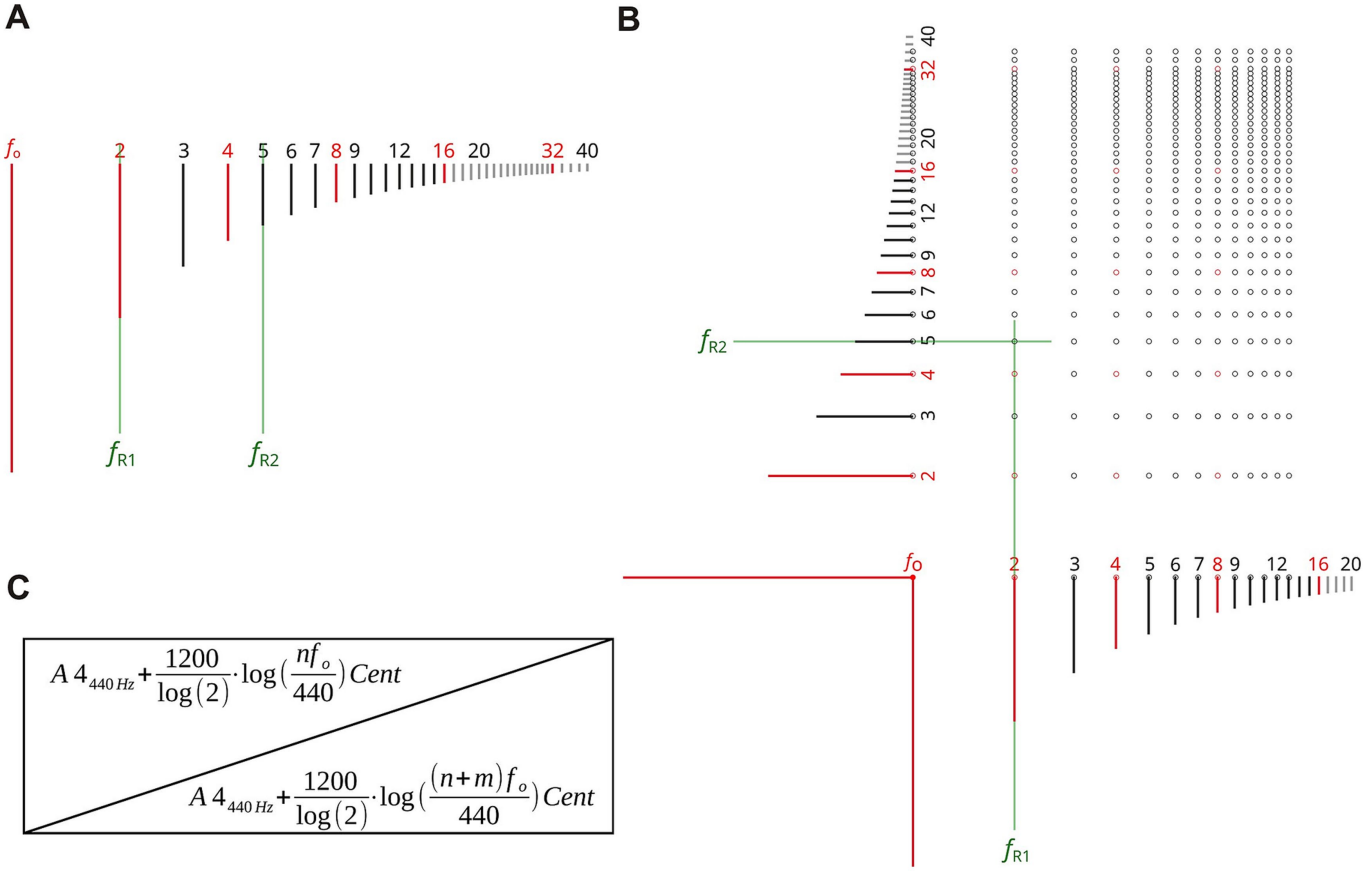
Diagram of vowel resonances. Illustration of a harmonic series in which the two vowel resonances align precisely with harmonics of the fundamental frequency, termed here as harmonic vowel. The first vowel resonance corresponds to the second harmonic (f_R1_ = 2f_o_), and the second resonance to the fifth harmonic (f_R2_ = 5f_o_), exemplifying a harmonic relationship **(A)**. Matrix of pairs of harmonics. The marked point illustrates the configuration shown in **(A)** in a two-dimensional grid, where f_R1_ meets the second harmonic and f_R2_ the fifth harmonic of the singing tone **(B)**. Matrix of harmonic vowel resonance pitches as a function of the fundamental frequency f_o_. Resonance frequencies are displayed logarithmically as pitch values in cents relative to A4 **(C)**.

In the proposed model, the resonance frequency pairs *f*_R1_ and *f*_R2_ of each vowel correspond to specific harmonics (n*f*_o_) in the voice spectrum, forming integer harmonic frequency ratios with the natural harmonic series. As illustrated in Figure 1A, such configurations—where both resonance frequencies coincide with integer multiples of the fundamental frequency *f*_o_—are referred to as “harmonic vowels”. In contrast, “non-harmonic vowels” occur when one or both vowel resonances do not align with partials, i.e., when their frequencies do not form integer ratios with *f*_o_. Such conditions are typically associated with reduced vocal brilliance and diminished resonance efficiency.

Each sung pitch defines a specific set of possible harmonic vowel configurations. Only those combinations that fall within the physiologically plausible range of human vowel resonances are considered relevant for singing.

To systematically represent these combinations, a matrix was constructed by plotting the harmonics aligned with the first vowel resonance (R_1_) along the x-axis and those aligned with the second resonance (R_2_) along the y-axis (Figure 1B). Each cell in the matrix thus indicates a pair of harmonics capable of simultaneously exciting both vowel resonances. Because musicians usually work in terms of pitch rather than frequency, the matrix was adapted accordingly: resonances are displayed both as logarithmic frequency values and as corresponding musical pitch labels (Figure 1C) (Formula 1).


(1) $$\begin{array}{l} H_{n,m≔({\mathrm{nf}}_{o},(n+m)f_{o})\;\;\;\;\;\;\;\;\;\text{for}\;\;\;n\in }\{1,...,N\},m\in \{0,...,M\}\\ H=(\begin{array}{cccc} \left(1f_{o},1f_{o}\right) & \left(1f_{o},2f_{o}\right) & \cdots & \left(1f_{o},(1+M)f_{o}\right)\\ \left(2f_{o},2f_{o}\right) & \left(2f_{o},3f_{o}\right) & \cdots & \left(2f_{o},(2+M)f_{o}\right)\\ \vdots & \vdots & \ddots & \vdots \\ \left({\mathrm{Nf}}_{o},{\mathrm{Nf}}_{o}\right) & ({\mathrm{Nf}}_{o},(N+1)f_{o} & \cdots & \left({\mathrm{Nf}}_{o},(N+M)f_{o}\right) \end{array}) \end{array}$$

#### Limits of vowel resonances

The physiological structure of the vocal tract imposes constraints on the frequency range within which vowel resonances can occur. Only those pairs of harmonics that lie within the articulable range of the vocal tract are viable for resonance. For the construction of the Singing Phonetics Diagram, three boundary conditions were defined:

1. *Resonance ordering:* The second resonance frequency (*f*_R2_) must consistently be higher than the first (*f*_R1_), such that *f*_R1_ < *f*_R2_.
2. *Physiological frequency ranges:* Based on empirical observations by the first author, the typical resonance ranges for vowels extend approximately from B3 to C6 for R_1_, and from E4 to F7 for R_2_. These values are approximate and may vary across individuals and voice types (e.g., children, women, men).

- R_1_: 240 Hz ⪅ *f*_R1_ ⪅ 1,000 Hz (≈ B3 − C6)
- R_2_: 320 Hz ⪅ *f*_R2_ ⪅ 2,700 Hz (≈ E4 − F7)

1. *Vowel triangle constraint:* As *f*_R1_ increases, the variability of *f*_R2_ decreases. The maximum variability of *f*_R1_, on the other hand, is observed at intermediate *f*_R2_ values (around E6 ≈ 1,300 Hz) and decreases toward higher and lower *f*_R2_. This interdependent dynamic reflects the structure of the classical acoustic-phonetic vowel triangle.

**Figure 2:**
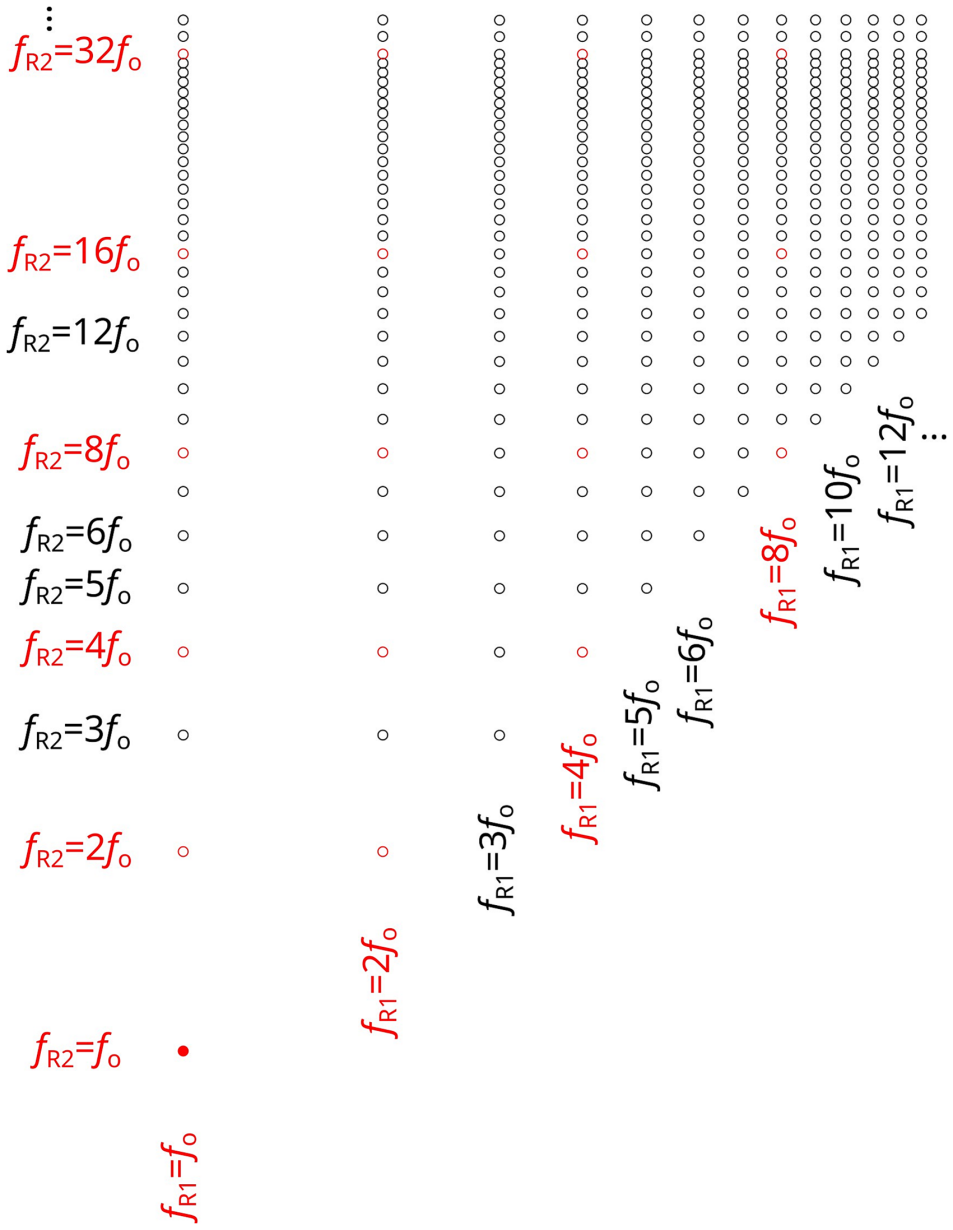
Harmonic matrix. Each point represents a pair of harmonics (f_R1_ = nf_0_, f_R2_ = mf_0_) capable of simultaneously exciting the two vowel resonances. Only frequency pairs above the diagonal (f_R2_ > f_R2_) are considered physiologically relevant, corresponding to articulable harmonic vowel configurations.

Only frequency pairs that satisfy these three conditions—particularly the ordering condition—are included in the matrix of viable harmonic combinations. Graphically, this is represented by retaining only the entries located above the diagonal line (*f*_R2_ > *f*_R1_) in the matrix (see Figure 2).

#### The singing phonetics diagram

**Figure 3:**
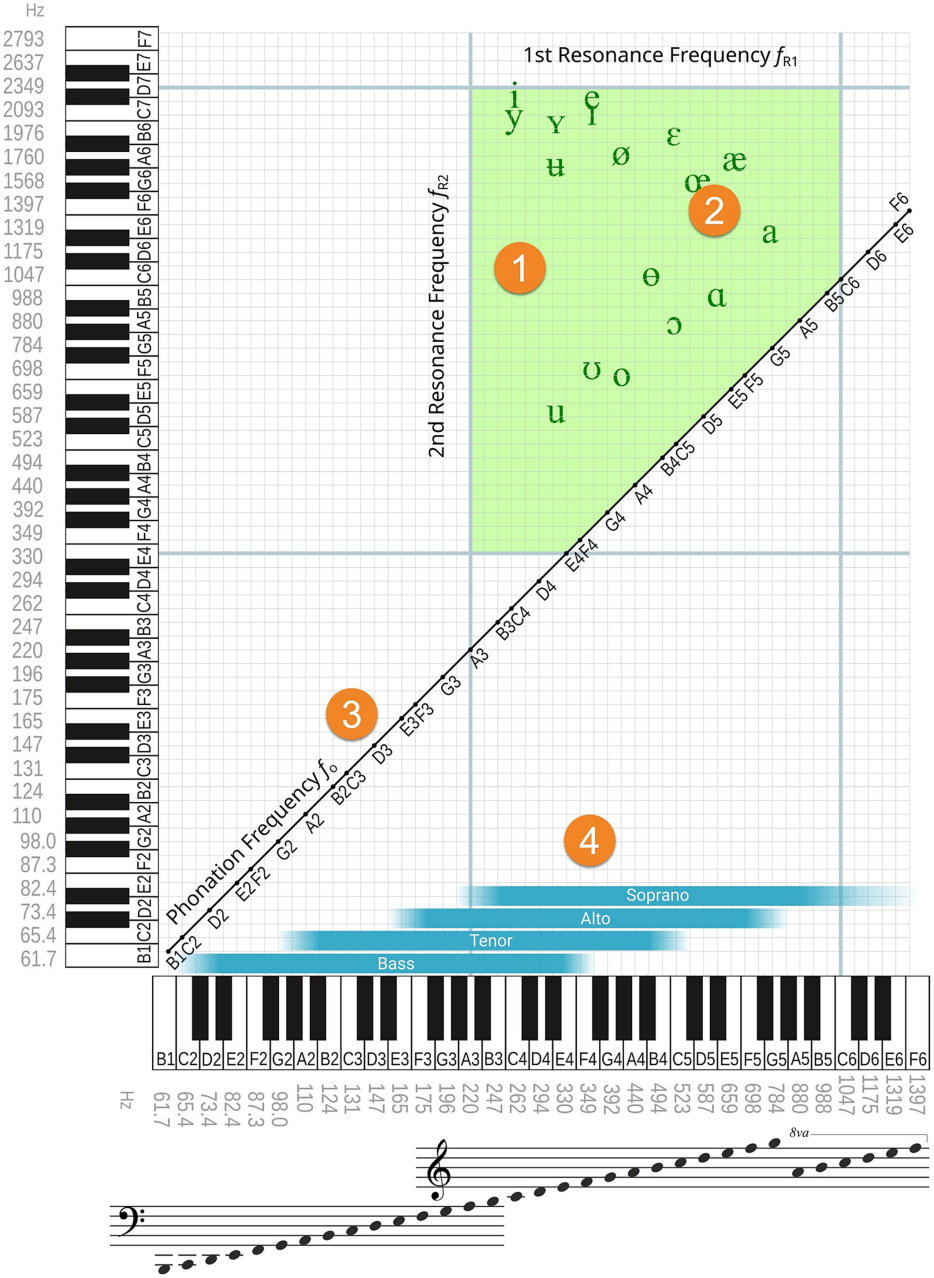
Singing phonetics diagram. Acoustic-phonetic vowel triangle embedded within a musical-acoustic framework, displaying resonance frequencies of the vocal tract as pitch values. (1) Shaded green area: physiologically plausible resonance range of the vocal tract (*f*_R1_, *f*_R2_). (2) Vowel triangle based on phonetic data from Swedish (Fant et al., 1969), with symbols indicating approximate articulatory positions. (3) Diagonal line: fundamental pitch (*f*_0_) as reference for sung tones. (4) Blue bands: typical singing ranges across voice types, aligned with corresponding f₀ values.

The foundational layer of the diagram shown in Figure 3 situates the classical acoustic-phonetic vowel triangle within a musical framework, representing vowel resonance frequencies as musical pitches.

Field (1) approximates the articulatory resonance range of the human vocal tract, with *f*_R1_ spanning from A3 to C6 and *f*_R2_ from E4 to E7. On average, resonance frequencies in the female vocal tract are approximately a minor third higher than in the male vocal tract, reflecting anatomical differences in laryngeal size and vocal tract length. This region also includes a vowel triangle marked with phonetic symbols, here drawn from Swedish (Fant et al., 1969), a language known for its extensive vowel inventory. The use of Swedish phonetic signs is illustrative and can be replaced with those from any language.

However, in singing, the range of articulatory configurations extends beyond speech norms, approaching the physiological limits of vowel production. As such, phonetic symbols serve only as approximate indicators of timbre in the singing context, particularly at higher pitches where vowel perception changes significantly (Maurer et al., 2020). Traditional vowel triangles become less effective at higher pitches, especially above C5, where phonetic symbols often no longer correspond reliably to the perceived timbre of sung vowels.

To contextualize resonance within vocal production, the harmonic matrix (Figure 2) is superimposed onto the Singing Phonetics Diagram. Each fundamental frequency *f*_o_ aligns with the corresponding sung pitch along the diagonal, offering a clear overview of how harmonics interact with vocal tract resonances across the vowel triangle space. Points within the matrix that fall within the physiologically plausible resonance field represent potential harmonic vowels—those configurations capable of maximizing resonance.

The diagram highlights how adjustments of the vocal tract—initially guided by speech-based vowel categories—can be refined to optimize singing resonance across a wide pitch range. This becomes especially relevant in the upper register, where traditional phonetic markers fail to predict perceived vowel quality.

#### Illustrative scenarios

Two exemplary scenarios underscore the utility of the Singing Phonetics Diagram in demonstrating how articulatory adjustments—originally derived from speech vowel categories—can be optimized for singing resonance at different pitch ranges.

##### Example 1: enhancing resonance in the lower range

**Figure 4:**
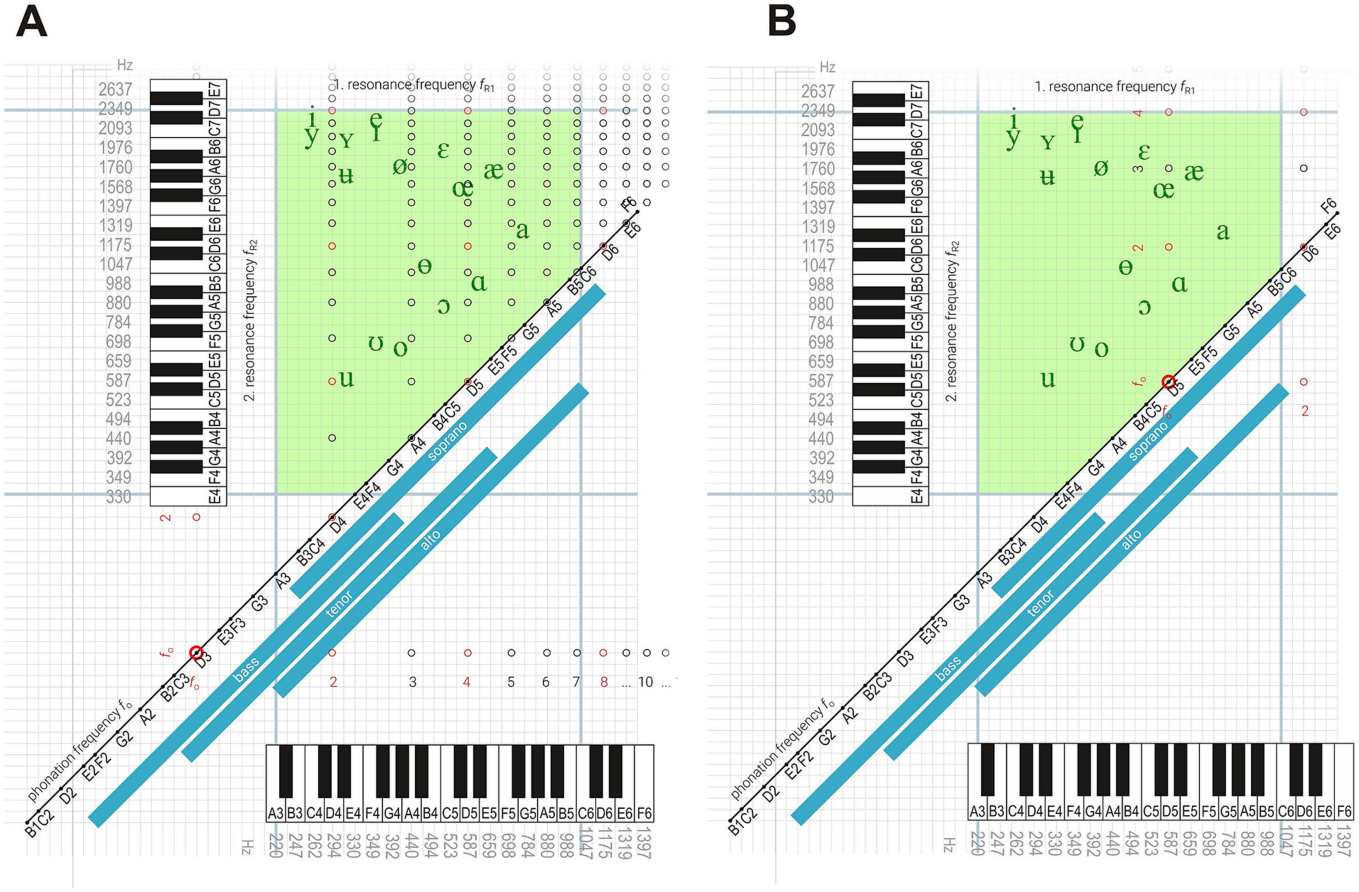
Harmonic vowel patterns within the vowel triangle. Harmonic matrix illustrating the distribution of harmonic vowel configurations for the fundamental pitch D3. The alignment of partials with vowel resonances reveals several possible harmonic vowels within the vowel triangle **(A)**. Distribution of partials for the fundamental pitch D5. Only two vowel positions within the vowel triangle simultaneously activate both vowel resonances, highlighting the resonance limitations in the upper singing range **(B)**.

Consider the harmonic vowel configurations for the fundamental tone D3, which lies below the typical resonance range of the vocal tract (Figure 4A). When the vocal tract is shaped toward vowels between [u] and [i], of the vocal tract (Figure 4A). When the vocal tract is shaped toward vowels between [u] and [i], the second harmonic (D4) aligns with the first resonance, *f*_R1_ = 2 *f*₀, resulting in increased amplitude of that harmonic.

Within this vowel space, fourteen harmonic vowels can be formed where the second resonance (*f*_R2_) aligns with partials ranging from 3*f*₀ to 16*f*₀, producing distinct timbral nuances. Most of these lack corresponding phonetic symbols because their acoustic specificity exceeds what is required in spoken language. When *f*_R1_ *=* 3 *f*₀, another set of twelve harmonic vowels emerges ([o]–[ø]), and with *f*_R1_ *=* 4*f*₀, eight harmonic vowels ([ɑ]–[œ]) are possible. The upper limit of *f*_R1_ is reached at C6 (*f*_R1_ = 7*f*₀), where only two open vowel qualities remain, corresponding to *f*_R2_ *=* 8 *f*₀ (D6) and *f*_R2_ *=* 9 *f*₀ (E6).

##### Example 2: resonance constraints in the upper range

For a fundamental pitch of D5, which falls within the upper end of the *f*_R1_ range, the typical vowel tract shapes for speech vowels like [u], [o], or [e] do not allow for effective excitation of *f*_R1_ (Figure 4B). Nevertheless, *f*_R2_ can still be excited by the second, third, or fourth harmonic. A professionally trained soprano articulating [i] at this pitch often modifies the vocal tract toward configurations resembling [ɛ] or [æ] to reduce the excessive brightness and acoustic sharpness associated with [i].

Only two vowel configurations at D5 can effectively excite both resonances simultaneously:

- (*f*_R1_ = *f*₀, *f*_R2_ = 2 *f*₀): corresponding to a timbre ranging from [ɑ]–[œ] and [ɵ]–[a].
- (*f*_R1_ = *f*₀, *f*_R2_ = 3 *f*₀): corresponding to a timbre ranging from [œ], [ɛ], and [æ].

All other configurations fail to excite both resonances simultaneously.

These examples highlight how targeted vocal tract adjustments—guided by the diagram—can optimize resonance and thus vocal quality across the singing range.

#### Practical applications

The Singing Phonetics Diagram offers a range of practical benefits. Foremost, it provides a physically accurate and language-independent representation of vocal resonance phenomena. By mapping partial–resonance interactions visually, the diagram enables singers and teachers to identify effective articulatory strategies for enhancing or attenuating specific harmonics, thus enabling deliberate control over vocal timbre.

A particularly valuable application lies in training singers to tune their vowel resonances to pitch. Each matrix position corresponds to a precisely controllable articulatory configuration. A prerequisite for this is overtone listening—the ability to perceive resonance frequencies as distinct pitch elements (Saus, 2017b; Schneider et al., 2005a, 2005b). As early as the mid-19th century, Hermann von Helmholtz described the perceptibility of resonances as pitches (von Helmholtz, 1857, 1859, 1896). Although rarely trained in Western vocal pedagogy, this ability can be acquired within minutes (Saus, 2017b), and overtone singing techniques allow singers to develop semitone-level resonance control within days (Saus, 2017a, 2021).

Well-trained singers can actively adjust each resonance point within the matrix: R_1_ is primarily modulated through jaw and mid-tongue curvature, while R_2_ is largely controlled by the tongue’s pharyngeal portion (Neuschaefer-Rube et al., 2002; Richter et al., 2017).

Originally developed for use in choral phonetics, the diagram has proven valuable in choral intonation training (Saus, 2009b, 2015). Choir directors can select vowels that align R_2_ with shared partials across different voices, thereby enhancing intonation purity and ensemble blending.

Because the diagram is resonance-strategy agnostic, it can also be used to avoid resonance where necessary—for instance, when full resonance may interfere with glottal efficiency at certain dynamics (Titze, 2008; Titze and Worley, 2009).

Finally, existing resonance strategies—such as those by Bozeman (2013), Bozeman and Spivey (2021), Coffin (1980), Miller and Schutte (1990), or Stockhausen’s vowel square in Stimmung (Stockhausen, 1968; Saus, 2009a)—can be mapped onto the diagram and systematically compared.

The diagram has been licensed to Sygyt Software and is now integrated into the VoceVista Video analysis software (Maass et al., 2004). This implementation enables playback of synthetic vowel sounds based on selected resonance pairs—serving as highly effective templates in vocal pedagogy.

Despite its potential, pedagogical use of the diagram has revealed that only a minority of singers spontaneously perceive vowel resonances as pitches. This underscores the importance of explicit training in overtone perception for effective use of resonance-based vocal strategies.

## Materials and methods

### Auditory tests

#### Harmonic awareness training (HAT)

To gain a deeper understanding of how singers differentially perceive sung vowels, resonances, and harmonics, it is essential to elucidate the underlying cognitive mechanisms.

The Harmonic Awareness Training (HAT) was originally designed by the first author as a program for singers in order to specifically acquire the ability to perceive vowel resonances or the harmonics emphasized by them as pitches. The HAT consists of 6 conditions (segments) of phone sequences sung at a constant pitch (D3) and designed such that the second vowel resonance, R_2_, always aligns with partials 8 through 12, producing the melody of Beethoven’s *Ode to Joy*: 10-10-11-12-12-11-10-9-8-8-9-10-10-9-9, etc. With the fundamental tone D3, *f*₀ = 147 Hz, the resulting resonance frequencies are as follows:

*f*_R2_ = 8 *f*₀ = 1,175 Hz = D6
*f*_R2_ = 9 *f*₀ = 1,321 Hz = E6 + 4c
*f*_R2_ = 10 *f*₀ = 1,468 Hz = F#6-14c
*f*_R2_ = 11 *f*₀ = 1,615 Hz = G#6-49c
*f*_R2_ = 12 *f*₀ = 1762 Hz = A6 + 2c.

In each segment, specific aspects of speech timbre are added to or removed from the sound sequences. The syllable sequences created by adding consonants are designed to remain nonsensical and devoid of semantic meaning, thereby directing listeners’ focus to the harmonic components of the sound and ultimately enabling them to recognize the melody in each segment. Most listeners will not perceive the melody upon their first exposure to segment 1.

**Figure 5:**
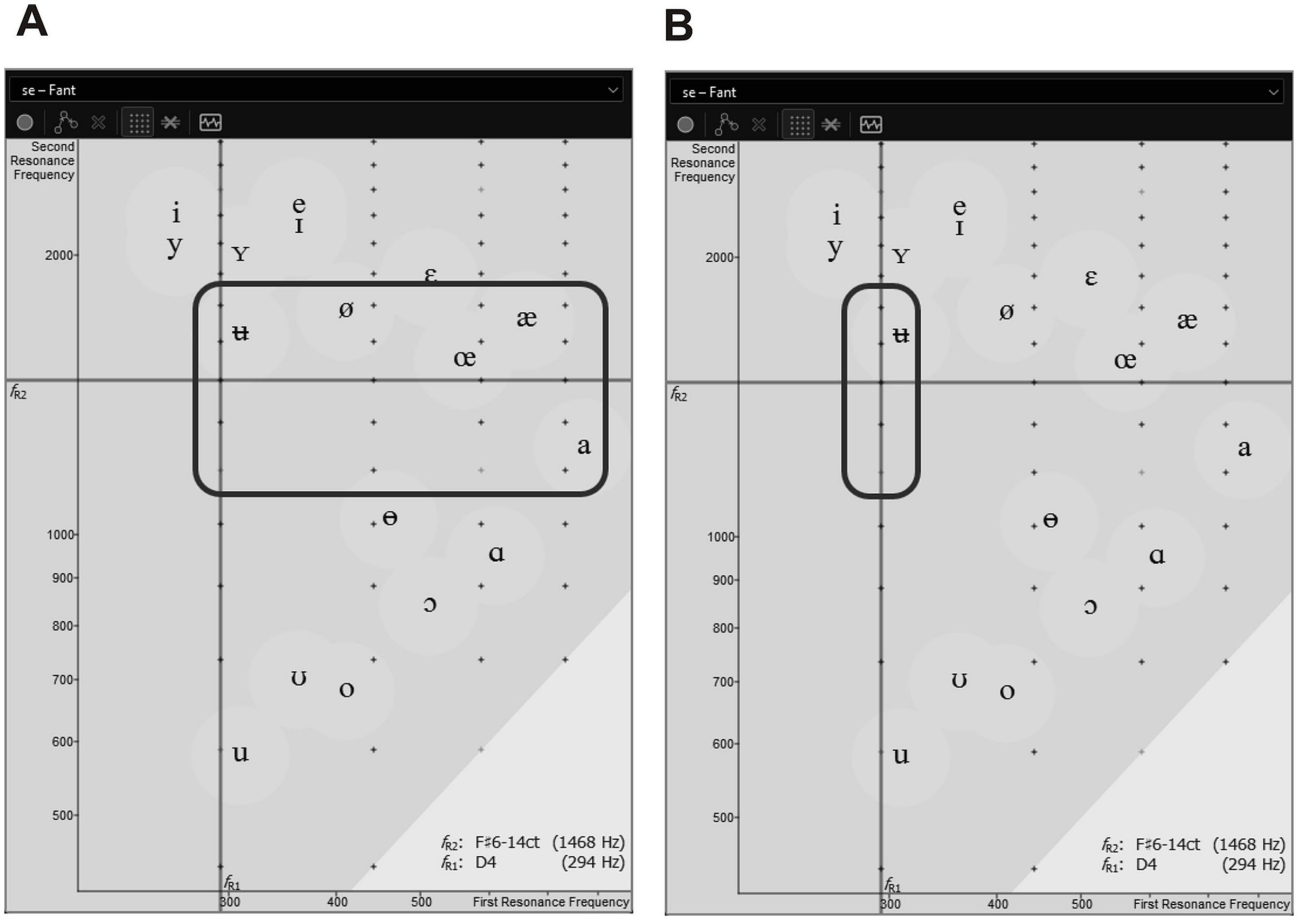
Vowel configurations in the HAT test across phone sequence segments. Vowel positions in segment 1 of the phone sequences. The second resonance (f_R2_) tracks harmonics 8 through 12 along the melody, while the first resonance (f_R1_) varies across harmonics 2 to 5, producing a broad range of vowel qualities **(A)**. Vowel positions in segments 2 to 6 of the phone sequences. While f_R2_ continues to follow partials 8 through 12, f_R1_ remains fixed on partial 2, resulting in a narrow vowel range centered around [ʉ]–[ʏ] **(B)**.

Six distinct segments were designed:

1. In the first segment, the first vowel resonance (*f*_R1_) was varied using vowels from the area marked in Figure 5A, accompanied by both voiced and voiceless consonants.
2. The second segment features *f*_R1_ fixed at 2 *f*₀ on the second harmonic using vowels from the area marked in Figure 5B, again with both voiced and voiceless consonants.
3. In the third segment, vowels from Figure 5B are paired with the consonants [d] and [n], while the fourth segment combines vowels from the same region with the nasal consonant [n].
4. The fifth segment uses only vowels from Figure 5B without any consonants, and includes a constriction of the ventricular folds.
5. Finally, the sixth segment varies the second resonance (R_2_) in a manner analogous to the vowels from Figure 5B, without any consonants, and employs an overtone singing technique to produce double resonances (*f*_R2_ = *f*_R3_).

The auditory training involves listening to the sequence of these segments in descending order—from segment 6 to segment 1—until the melody in segment 1 is recognized with certainty.

#### Pitch perception preference test (PPPT)

The auditory system can perceive either an actual or a missing fundamental frequency (*f*₀) within a sound spectrum and is even capable of perceptually restoring an absent fundamental. Additionally, it can consciously perceive the overtones in the frequency spectrum (fSP) by directing attention to them. The former ability, perceiving the present or missing fundamental frequency (“abstract listening”), is primarily associated with left-hemisphere function, whereas the latter ability, perceiving overtones (“concrete listening”), is mainly supported by the right hemisphere of the brain (Schneider et al., 2005a, 2005b). Here, “perceiving the fundamental frequency” means that listeners either hear a fundamental tone that is physically present in the spectrum or perceptually reconstruct it when it is absent. The PPPT (Schneider et al., 2005a, 2005b; Schneider and Wengenroth, 2009) includes 144 different pairs of harmonic complex tones. Each pair consists of two consecutive tones (duration: 500 ms, 10 ms rise-fall time, interstimulus interval 250 ms). Each test tone includes two, three, or four adjacent harmonics, omitting the fundamental frequency (Schneider et al., 2005a, 2005b). For each individual, an “index of pitch perception preference (PPPT index)” *δ* = (fSP − *f*₀)/(fSP + *f*₀) was computed (fSP: number of perceived spectral pitches, *f*₀ number of perceived fundamental pitches) ranging from +1 (only spectral pitch perception) to −1 (only fundamental pitch perception). Based on their PPPT scores, subjects were classified according to their predominant listening mode as “fundamental listeners” (*f*₀) if their scores ranged from −1 to 0, or as “spectral listeners” (f_SP_) if their scores were greater than 0 up to +1.

The stimuli were presented binaurally using an RME Hammerfall DSP Multiface system and closed dynamic headphones (Sennheiser HAD 200) designed for high-quality hearing tests. These headphones provide about 30 dB of passive attenuation in the frequency region of the stimuli used. The intensity was controlled not to exceed 75 dB SPL.

### Magnetoencephalographic (MEG) experiment

#### HAT-paradigm adapted for MEG

**Figure 6:**
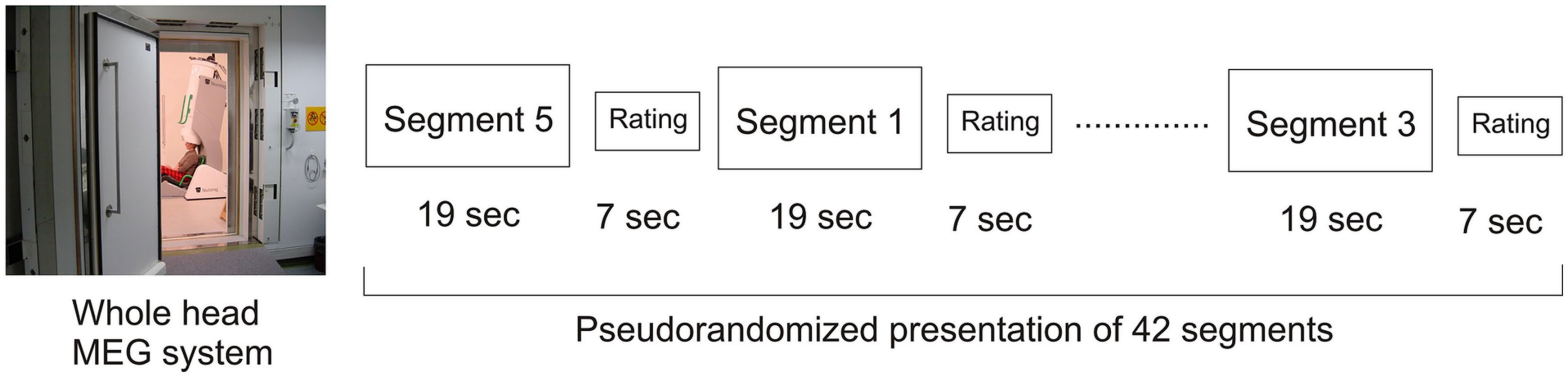
Experimental design of the HAT paradigm adapted for MEG. During the MEG measurement, the six different conditions (segments) of the HAT test were presented in a pseudorandomized order. Each segment lasted 19 s and consisted of a differently weighted mixture of semantically meaningless syllables and overtone melodies, followed by a 7-s interstimulus interval for subjective rating on a scale from 0 (“only speech-like syllables”) to 10 (“only overtone melody”). In total, 42 segments were presented while neural responses were recorded using a 122-channel whole-head MEG system.

Stimuli were parametrically arranged in six steps along a perceptual speech–song gradient, ranging from highly speech-like to purely musical. In the following, the term “speech content” is used to describe semantically empty syllables with phonetic characteristics of spoken language, designed to mimic speech-like input without conveying meaning. The individual sound segments were taken unchanged from the website version of HAT by W. Saus, presented each seven times in a pseudorandomized order (Figure 6, Saus, 2017c). Each of the six segments had a duration of 19 s, followed by a varying interstimulus interval of 7–7.5 s. The total duration of the MEG session was 18.3 min. The loudness was adjusted to a uniform intensity level of 70 ± 2 dB SPL using a Brüel & Kjaer artificial ear (type 4152) and a 2 cc coupler.

#### Participants

Seventeen experienced overtone singers (mean age: 46.5 ± 3.4 years, range: 19–65 years, 8 males and 9 females) participated in the study. The participants were classified as experienced overtone singers based on self-reported overtone singing practice and their individual Index of Musical Practice (IMP; Schneider et al., 2023). The IMP quantifies cumulative musical training as the product of weekly practice hours and years of training, combining formal instruction (e.g., in school or conservatory) and private practice time, following the method described in Bücher et al. (2023). An IMP value of 100 serves as a reference point for musically experienced middle-aged adults (Benner et al., 2017). In our sample, the mean IMP was 116.9 h/week × years (range: 25–340), indicating substantial musical engagement. The group included six professional musicians and eleven advanced amateur musicians, all with overtone singing experience. Although the sample size was limited (n = 17), it was constrained by the fixed enrollment of the specialized overtone singing course during which the study was conducted. Nevertheless, large effect sizes (partial η^2^) observed across analyses suggest sufficient sensitivity to detect the neural effects of interest. All participants provided informed consent, and the study was conducted in accordance with the Declaration of Helsinki. Participants had no history of neurological or hearing impairments.

#### MEG recordings, preprocessing and data analysis

Neural responses to the auditory stimuli were recorded using a 122-planar gradiometer whole-head MEG system (Neuromag-122, Hämäläinen et al., 1993).

Before the recording session, four reference coils were attached to the participant’s head (left/right temples and left/right mastoids) using skin-friendly adhesive tape. An electronic digitizing pen was used to mark three anatomical landmarks (nasion, left and right preauricular points) to establish the head coordinate system. Additionally, 32 extra points on the head surface were digitized to improve co-registration accuracy.

During the MEG recordings, participants were seated comfortably under the MEG dewar in a sound-attenuated chamber. Stimuli were presented binaurally via 90 cm plastic tubes connected to foam ear pieces inserted into the ear canal. Shielded transducers housed in boxes next to the chair ensured minimal external noise interference. Participants were instructed to remain still and focus on the stimuli. They were instructed to rate their perception of each segment on a 0–10 salience scale, where 0 indicated “only speech-like syllables” and 10 indicated “only overtone melody” (Figure 6).

MEG signals were recorded at a sampling rate of 1,000 Hz, corresponding to a low-pass filter of 330 Hz (filter range: DC–330 Hz). Artifact correction and preprocessing were conducted using BESA Research 6.0 (MEGIS Software GmbH, Graefelfing, Germany). Data quality was assessed with the Event-Related Field (ERF) module, excluding noisy channels (3–7 per participant) and epochs exceeding a gradient of 600 fT/cm × s or amplitudes outside the range of 100–3,000 fT/cm.

Signal analysis was performed relative to a 100 ms pre-stimulus baseline. Fast Fourier Transform (FFT) analysis was used to calculate the spectral power of the neural responses (delta: 0–4 Hz, theta: 4–8 Hz, alpha: 8–13 Hz, beta: 13–30 Hz, and gamma: 30–50 Hz bands) separately for each stimulus segment, using a standard single-sphere head model (Hämäläinen and Sarvas, 1987; Scherg, 1990).

Grand averages were calculated across all participants (in total 42 artifact-free epochs per participant, i.e., over 7 epochs for each of the six segments) in a time window of 100 ms pre-stimulus to 11,000 ms post-stimulus.

**Figure 7:**
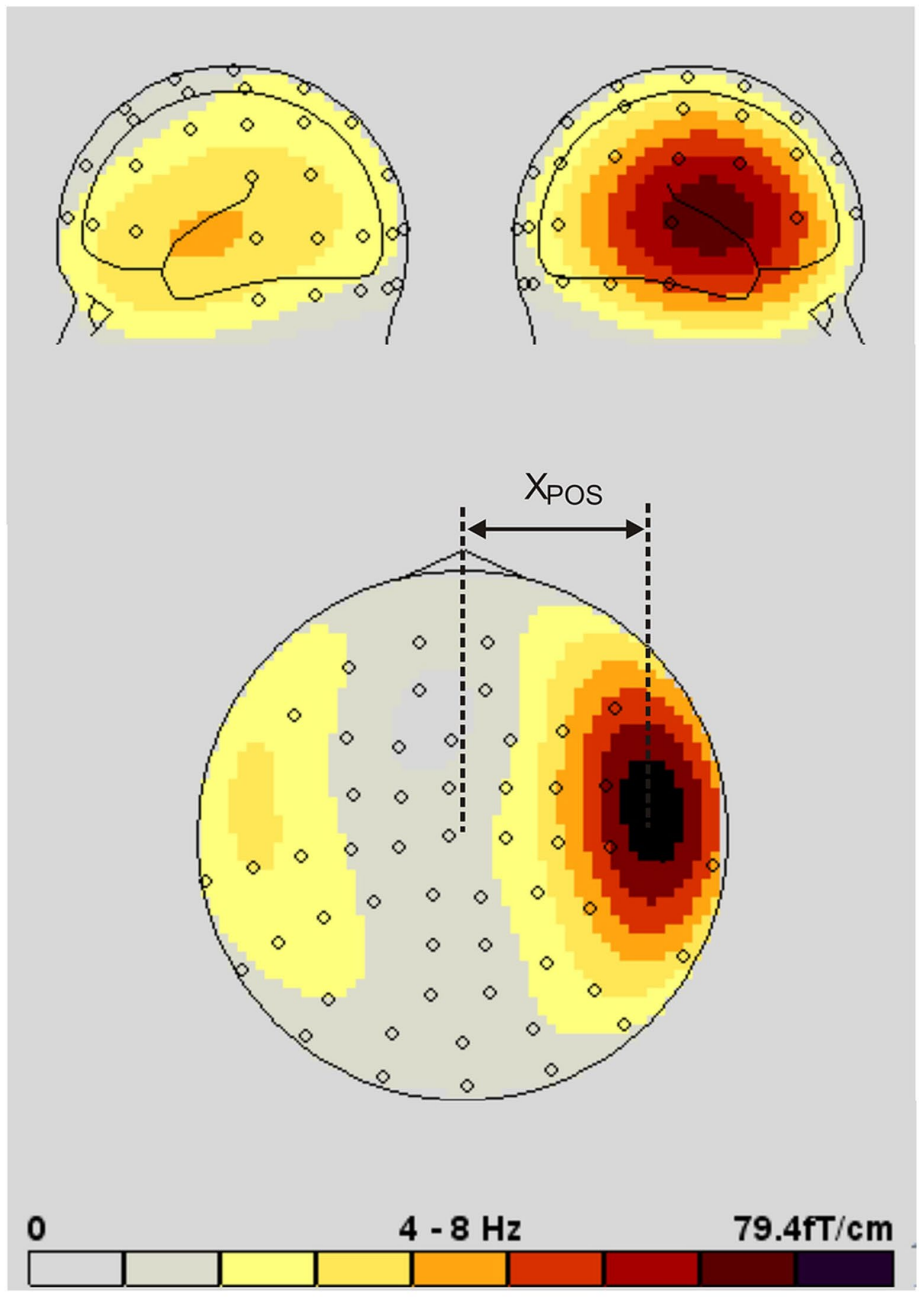
Topographic MEG map of auditory activity in the 4–8 Hz range. Exemplary brain topography illustrating the determination of hemispheric lateralization. The lateralization index (X_POS_) was calculated individually based on the center of gravity of the activity distribution across hemispheres, as indicated in the map.

Neural activity was quantified by measuring the spectral power (FFT) within distinct frequency bands. Brain topography was computed individually across the entire cortical surface for each condition. Furthermore, the lateralization (x-coordinate) of the center of gravity of the topographic activation map exhibiting the highest spectral power was determined (Figure 7). Due to the spatially extended nature of the oscillatory activity and the primary interest in lateralization and amplitude patterns, analyses were conducted at the sensor level. In contrast to EEG, MEG provides a higher spatial fidelity even without source modeling, which was therefore not pursued.

Additionally, we investigated the relationship between overtone-driven brain processing and stable individual auditory profiles for the perception of harmonic complex tones, as assessed by the PPPT (Schneider et al., 2005a, 2005b). We hypothesized that learning to perceive vowel resonances as distinct pitches would be reflected in changes of brain activation, particularly in frequency bands associated with auditory processing and sensory integration.

### Statistical analysis

For each of the five frequency bands considered (delta, theta, alpha, beta, gamma), separate one-way repeated-measures ANOVAs were conducted on the source-analysis parameters “frequency band power” (FBP) and “x-coordinate position” (X_POS_). X_POS_ reflects both the degree (i.e., distance from zero) and the direction (negative: left, positive: right) of hemispheric lateralization of the cortical sources identified in each frequency band, where zero corresponds to the sagittal (z) plane of the head. The within-subject factor was “stimulus type”, which systematically varied the perceived predominance of speech versus overtone content on a scale from 1 (syllables most prominent) to 6 (pure overtone singing).

In addition, the subjective ratings of the presented stimuli along the salience (Figure 6) were analyzed using a two-way ANOVA with the within-subject factor “stimulus type” and the between-subject factor “listening mode” (“fundamental listeners” [f_0_] vs. “spectral listeners” [f_SP_]).

In line with SPSS’s general linear model framework, the degrees of freedom for the within-subject effects were calculated based on the number of factor levels and the subject × condition error structure. Sphericity was assessed using Mauchly’s test for each ANOVA separately, and Greenhouse–Geisser corrections were applied where necessary. All *post hoc* comparisons following the ANOVAs were Bonferroni-adjusted.

Furthermore, to determine whether the mean X_POS_ values deviated significantly from the sagittal midline of the head—thus indicating hemispheric lateralization—one-sample *t*-tests were performed for each stimulus condition within each frequency band. To correct for multiple testing across the six stimulus conditions, Holm’s method was applied (Holm, 1979), a stepwise procedure that controls the family-wise error rate while maintaining statistical sensitivity. In addition, one-sample *t*-tests were performed on the average X_POS_ values across all six stimulus conditions, separately for each frequency band, to assess whether the cortical sources were generally lateralized toward a particular hemisphere for the stimulus material as a whole.

Supplementary correlational analyses were performed on interval-scaled data, using Pearson’s r when both variables were normally distributed, and Spearman’s rho (*ρ*) otherwise.

## Results

### Subjective perceptual salience of the stimulus material

**Figure 8:**
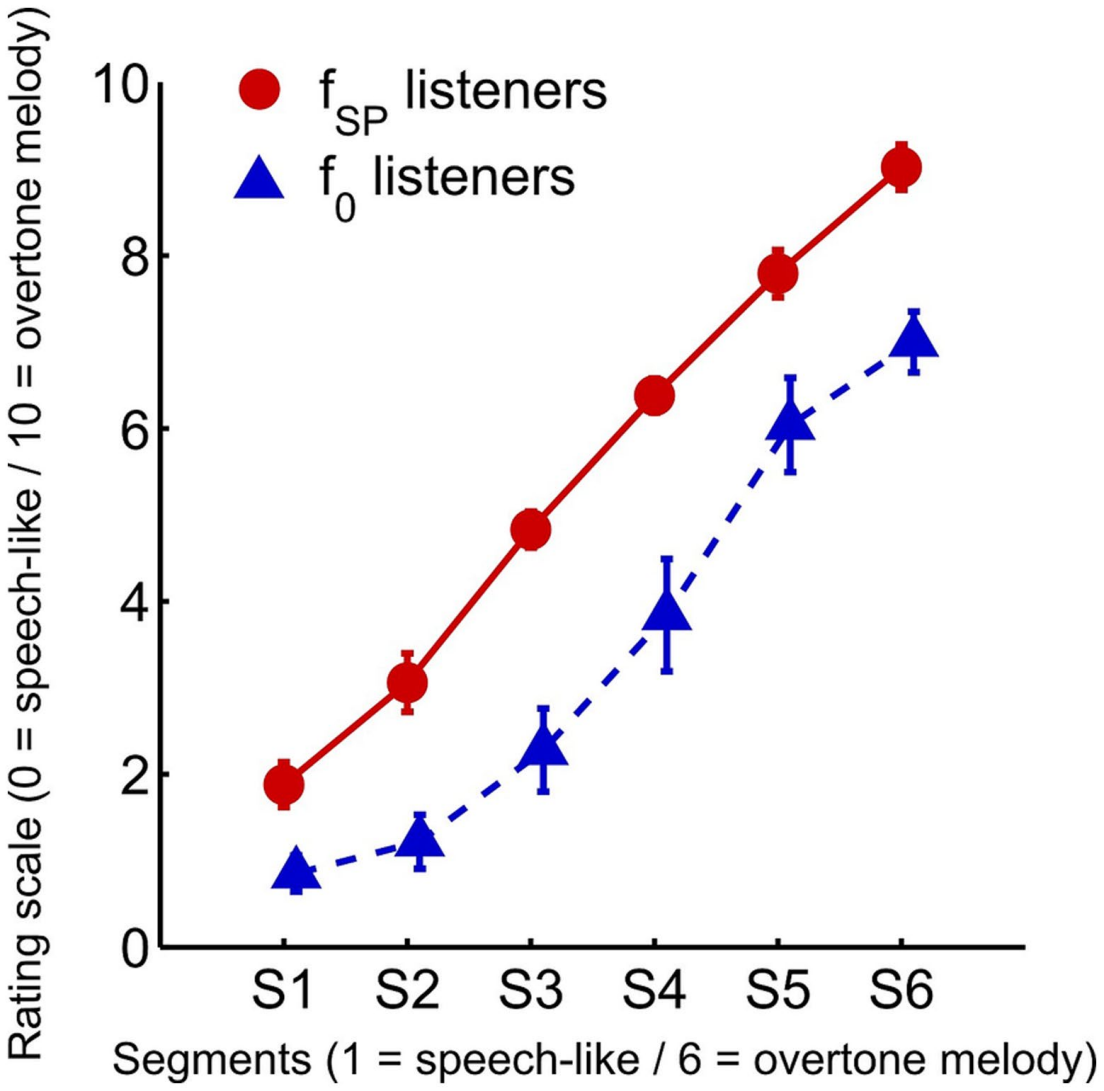
Subjective perception of speech–song continuum. Perceptual salience ratings (0–10) for the six stimulus segments of the HAT paradigm, shown separately for spectral pitch listeners (red circles, solid line) and fundamental pitch listeners (blue triangles, dashed line). Participants rated each segment based on how clearly they perceived the overtone melody (“Ode to Joy”), with lower ratings indicating speech-like syllables and higher ratings indicating overtone-dominant, song-like percepts. Error bars represent ±1 SEM.

With regard to the salience scale, the ANOVA revealed a highly significant main effect of “stimulus segment” (*F*(5,75) = 372.5, *p* = 1.9 × 10^−51^, partial η^2^ = 0.96). This demonstrates that the manipulation of the stimulus material from speech-like to overtone-dominant was remarkably effective, achieving near-perfect perceptual differentiation between conditions (Figure 8). Segments with reduced speech content and enhanced overtone salience (particularly segment 6, featuring double resonances) were consistently rated as more “song-like”, while segments with strong speech-like characteristics (particularly segment 1) received the lowest salience ratings.

### MEG findings

MEG analyses revealed significant effects of both FBP and hemispheric lateralization across frequency bands, as reflected in the ANOVA findings, depending on vowel resonance perception and the perceived speech content in the auditory stimuli.

#### Hemispheric lateralization

**Figure 9:**
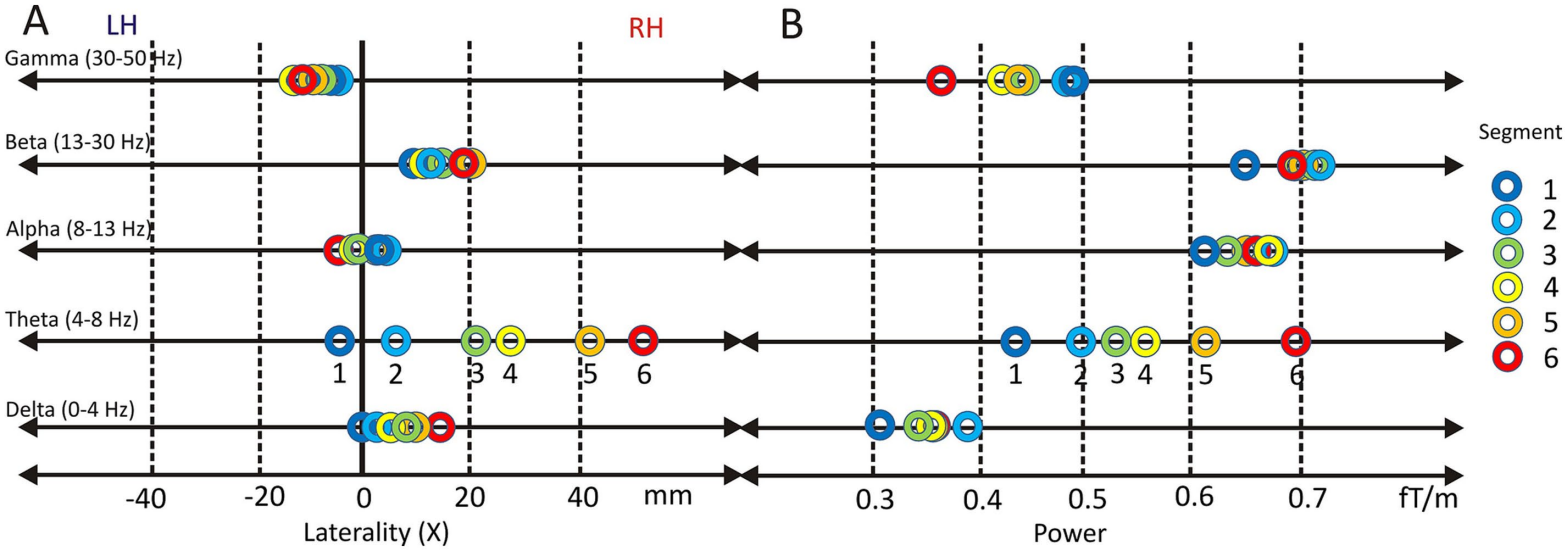
Lateralization and power of oscillatory brain activity across frequency bands during vowel resonance perception. Topographic center (X_POS_) across five frequency bands (Gamma, Beta, Alpha, Theta, and Delta) while listening to segments 1–6 of the Harmonic Awareness Training (HAT). Positive values indicate right-hemispheric lateralization (RH), negative values left-hemispheric lateralization (LH) **(A)**. Oscillatory power in the same frequency bands during the same segments, shown in femtoTesla per meter (fT/m) **(B)**.

In the following, the degree of FBP lateralization, as indicated by X_POS_ (negative: left-sided; positive: right sided), will be analyzed for each frequency band (Figure 9A).

##### Delta band

Across all conditions, delta-band activity was significantly lateralized to the right hemisphere (X_POS_ = 0.91 ± 1.2; t(16) = 3.15, *p* = 6.2E-3, Cohen’s d = 1.19). A repeated-measures ANOVA revealed a significant effect of speech-like content on X_POS_ (*F*(5, 80) = 4.78, *p* = 4.0E-3, partial η^2^ = 0.23), indicating that rightward lateralization increased systematically as speech-likeness decreased. *Post hoc* comparisons showed a significant difference between condition 1 (highest speech-like content; M = 0.15, SD = 1.54) and condition 6 (no speech-like content; M = 1.68, SD = 1.16). Holm-corrected one-sample *t*-tests confirmed significant right-hemispheric lateralization for conditions 5 (*p* = 1.3E-3) and 6 (*p* = 2.0E-5).

##### Theta band

The theta band exhibited pronounced right-hemispheric lateralization (X_POS_ = 2.4 ± 2.0). A repeated-measures ANOVA with Greenhouse–Geisser correction revealed a highly significant effect of stimulus type on theta-band lateralization (*F*(2.1, 34.1) = 70.88, *p* = 3.6E-13, partial η^2^ = 0.82), indicating that over 80% of the variance in X_POS_ was explained by decreasing speech content. Post hoc comparisons showed significant differences between all conditions except between conditions 3 and 4, reflecting a systematic rightward shift as overtone salience increased. Holm-corrected one-sample *t*-tests confirmed significant right-hemispheric lateralization relative to the sagittal midline for conditions 3 to 6. Across all stimuli, theta-band activity was significantly lateralized to the right hemisphere (t(16) = 4.97, *p* = 1.4E-4, Cohen’s d = 1.19).

##### Alpha band

The alpha band showed no evidence of systematic hemispheric lateralization (X_POS_ = 0.2 ± 1.5). Repeated-measures ANOVA revealed no significant effect of stimulus type on X_POS_, and Holm-corrected one-sample *t*-tests indicated no significant deviation from the sagittal midline in any condition. Accordingly, overall alpha-band lateralization was not significant (t(16) = 0.66, n.s.).

##### Beta band

Beta-band activity showed a consistent right-hemispheric lateralization across stimulus conditions (X_POS_ = 1.74 ± 1.5; t(16) = 4.74, *p* = 2.2E-4, Cohen’s d = 1.51). While the repeated-measures ANOVA revealed no significant effect of stimulus type on X_POS_, Holm-corrected one-sample *t*-tests confirmed significant rightward lateralization relative to the sagittal midline in all six conditions (all *p*-values between 8.3E-3 and 5.0E-2).

##### Gamma band

Gamma-band activity showed a moderate left-hemispheric lateralization overall (X_POS_ = −0.27 ± 1.4; t(16) = −2.67, *p* = 1.7E-2, Cohen’s d = 1.43). The repeated-measures ANOVA revealed no significant effect of stimulus type on lateralization. However, Holm-corrected one-sample *t*-tests indicated significant leftward deviations from the sagittal midline in conditions 3 (*p* = 9.8E-3) and 4 (p = 1.4E-3), likely reflecting reduced interindividual variability in these specific stimulus contexts rather than a consistent modulation by speech content.

#### Frequency band power

In addition to lateralization, the mean power of each frequency band (FBP) was analyzed across stimulus conditions (Figure 9B). No statistically significant effects were observed for delta-, alpha-, or beta-band power.

In contrast, the theta frequency band (mean FBP = 0.35 ± 0.13) showed a pronounced increase in power across stimulus conditions. A highly significant main effect of speech content was observed (*F*(5, 80) = 30.23, *p* = 3.97E-17, partial η^2^ = 0.654). Theta power increased progressively from condition 1 to condition 6 as speech content decreased and overtone salience increased (c1: 0.43 ± 0.16; c2: 0.49 ± 0.20; c3: 0.53 ± 0.23; c4: 0.56 ± 0.23; c5: 0.62 ± 0.26; c6: 0.69 ± 0.25). Bonferroni-corrected pairwise comparisons confirmed significant differences between most conditions, with the largest contrast observed between condition 1 and condition 6 (*p* = 1.2E-5).

The gamma frequency band (mean FBP = 0.44 ± 0.26) exhibited a moderately significant main effect of speech content (*F*(1.6, 26.3) = 3.63, *p* = 4.9E-2, partial η^2^ = 0.185). However, Bonferroni-corrected pairwise comparisons did not reveal statistically significant differences between individual stimulus levels.

In summary, these results demonstrate a clear linear increase in theta-band power as speech content decreases. Gamma-band activity showed a trend toward decreasing power with decreasing speech-content, although without consistent significant pairwise effects.

Notably, theta and gamma band power were negatively correlated (*ρ* = −0.53, *p* = 0.028), suggesting complementary functional roles of the two frequency bands in sound processing.

#### Influence of pitch perception preference

**Figure 10:**
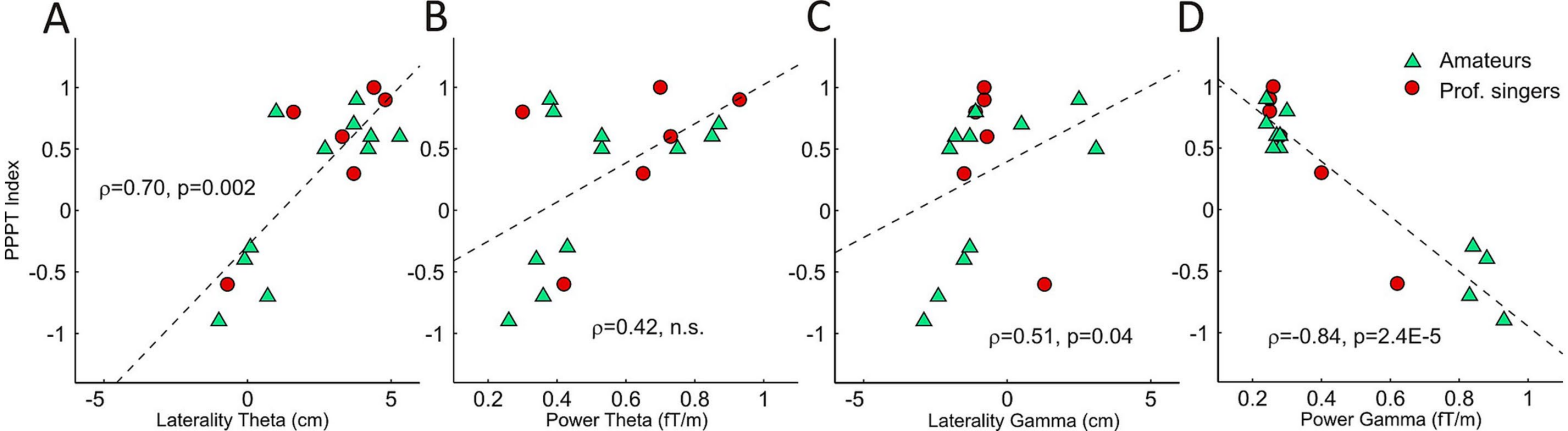
Correlation between the Pitch Perception Preference Test (PPPT) index and hemispheric lateralization (X_POS_) in the theta band. Higher values indicate stronger preference for overtone listening, which is associated with increased right-hemispheric lateralization **(A)**. Correlation between PPPT index and theta-band power (in fT/m), showing a positive but non-significant trend **(B)**. Correlation between PPPT index and lateralization in the gamma band. Overtone preference is linked to stronger right-lateralization **(C)**. Correlation between PPPT index and gamma-band power, showing a strong negative association. Fundamental pitch perception is associated with increased gamma power and left-hemispheric dominance **(D)**. Triangles represent amateur singers, circles represent professional singers.

As evident from Figure 10, the distribution of the PPPT index, ranging from −1 (maximal fundamental pitch perception) to +1 (maximal overtone perception), revealed two clusters. Five individuals exhibited negative values, classifying them as fundamental pitch listeners (f_0_), whereas twelve individuals showed positive values, classifying them as spectral listeners (f_SP_) (Figure 10A). Notably, no participant displayed values near zero, supporting previous findings (Schneider et al., 2005a, 2005b; Seither-Preisler et al., 2007) that pitch perception preference tends to follow a bimodal rather than a continuous distribution. The mean PPPT index of the participants in this study was 0.31 ± 0.63. With regard to the perceptual salience of the HAT stimuli, there was a clear difference between the two groups (*F*(1,15) = 44.7, *p* = 7.3 × 10^−6^, partial η^2^ = 0.75). f_SP_ listeners perceived all six stimulus segments as more overtone-dominant (5.5 ± 0.4) than f_0_ listeners (3.5 ± 0.8; see Figure 8).

Correlational analyses showed that the PPPT index was systematically associated with cortical FBP (Figures 10A–D). Negative correlations between the PPPT index and FBP indicate that a given cortical frequency band is related to fundamental pitch perception, whereas positive correlations indicate a relation to spectral overtone perception.

Strikingly, the PPPT index showed a strong negative correlation with gamma-band power (*ρ* = −0.84, *p* = 2.4e–5, Figure 10D), indicating a neural marker for the individual extent of fundamental pitch perception. The correlation with theta-band power was in the opposite direction (ρ = 0.42, *p* = 0.09), but did not reach statistical significance.

To examine whether this trait-like perceptual tendency is reflected in neural sensitivity to overtone salience, we correlated PPPT values with the response difference between stimulus level 6 (pure overtone singing) and level 1 (speech-like syllables masking the overtone content). A larger difference indicates greater neural responsiveness to the emergence of overtone structure.

For the theta band, this difference correlated positively with the PPPT index (ρ = 0.68, *p* = 2.8e–3), whereas for the gamma band, the correlation was negative (ρ = −0.56, *p* = 0.021).

This pattern demonstrates that f_SP_ listeners with a stronger predisposition toward overtone perception not only exhibited higher theta band power, but also a greater cortical responsiveness to the overtone-focused stimulus manipulation. The PPPT index was positively correlated with frequency band lateralization (X_POS_) both in the theta band (ρ = 0.70, *p* = 0.002, Figure 10A) and the gamma band (ρ = 0.51, *p* = 0.036, Figure 10C). Evidently, f_SP_ listeners were characterized by an increased right-hemispheric lateralization in both bands.

## Discussion

### From resonance theory to neural dynamics

This study presents a novel acoustic-phonetic framework that redefines vowel perception and production as a resonance-based phenomenon, in which vocal tract configurations selectively amplify harmonics. This approach is formalized in a pitch-based vowel diagram, which maps vocal tract shapes onto musical interval structures rather than articulatory categories. By aligning vowel formants with the overtone series, the model bridges articulation, spectral acoustics, and perceptual experience—offering a coherent framework particularly suited to fine-grained timbral vocalizations.

To explore whether these resonance-related principles are reflected in neural dynamics, we implemented a stepwise stimulus design in which the perceptual prominence of overtone components was systematically modulated across six distinct conditions (segments)—without altering the underlying harmonic spectrum. This manipulation induced a rapid, yet gradual perceptual transition from speech-like syllables that preserved key phonetic and phonological properties typical of spoken language to overtone-dominant stimulus segments. The participants’ precise differentiation of stimulus salience along the speech-like to overtone-dominant dimension validates the stimulus paradigm, indicating that the observed neural activation patterns were directly related to their subjective experience. Correspondingly, robust changes in brain oscillations reflected these perceptual shifts. Theta-band power increased progressively across the six stimulus levels toward the purely overtone-dominant condition, which contained no speech-like information, and this was accompanied by a consistent rightward shift in cortical activation. The magnitude of this effect was remarkable. Over 80% of the variance in lateralization was explained by stimulus type. This suggests a dynamic reconfiguration of activity in the auditory processing networks, transitioning from left-dominant, speech-related decoding to right-hemispheric representation of spectral features.

Our MEG data revealed a rightward lateralization of theta-band activity, whereas gamma-band activity showed a relatively stronger left lateralization. This pattern likely reflects complementary contributions of the two frequency bands to auditory processing and is consistent with the Asymmetric Sampling in Time (AST) model proposed by Poeppel (2003). The model suggests that the auditory cortices process sounds using distinct temporal windows in each hemisphere: left-hemisphere gamma-band oscillations (~30–50 Hz) capture rapid, segmental features, while right-hemisphere theta-band oscillations (~4–7 Hz) integrate slower, spectral, and prosodic information. The authors describe a nesting of gamma within theta activity that enables the dynamic organization of fast and slow acoustic cues, supporting coherent perception of speech and music. Extending the AST framework, Tóth et al. (2016) and Kocsis (2017) demonstrated that right-lateralized theta oscillations also increase during auditory stream segregation, indicating that theta-band dynamics contribute not only to temporal integration but also to the perceptual separation of concurrent sound sources. Importantly, our findings also align with recent spectro-temporal research emphasizing distinct neural encoding strategies for speech and music. Albouy et al. (2024) demonstrated that across cultures, speech and song are differentiated by specific spectro-temporal modulation patterns, with song characterized by slower temporal modulations and richer spectral detail. This dovetails with our observation of right-lateralized theta activity during overtone-dominant listening, potentially reflecting the processing of high-resolution spectral cues. Moreover, Albouy et al. highlighted a role for gamma-band activity in parsing fine-grained temporal fluctuations in speech, which is consistent with our left-lateralized gamma effects during more speech-like conditions. Integrating these perspectives suggests that overtone listening may engage neural networks specialized for precise spectral encoding, whereas speech-like perception draws on networks tuned to rapid temporal dynamics. Future work should directly link such spectro-temporal parameters in our stimuli with neural responses to further refine this interpretation.

The inverse relationship between gamma and theta dynamics observed in the present study suggests a functional complementarity: gamma-band activity appears relevant for the binding of single features into coherent auditory objects. In the musical domain, this applies both to harmonic binding at the level of the pitch of isolated complex tones and to the perception of simultaneous musical intervals with simple integer ratios. The former results in the subjective perception of a common fundamental pitch, while the latter contributes to the perception of musical consonance and the establishment of a tonal center, as highlighted by psychoacousticians and music perception researchers who described close relationships between virtual pitch, musical consonance, and tonality (Terhardt et al., 1984; Parncutt, 1988; Huron, 2001). There is converging evidence that these qualities are extracted from the fast temporal regularities of neural representations along the auditory pathway, from the auditory nerve to cortical areas (Krumbholz et al., 2003; Seither-Preisler et al., 2003, 2006a, 2006b), a process that has been linked to gamma-band oscillations and asymmetric temporal sampling (Schulte et al., 2002; Aksentijevic et al., 2013; Park et al., 2011; Poeppel and Assaneo, 2020).

In the language domain, gamma-band-related binding is essential for the identification and classification of speech-like elements characterized by rapid temporal fluctuations (Luo and Poeppel, 2007).

In a complementary manner, theta-band activity supports the precise identification of static or slowly changing acoustic features, such as the spectral pitches of individual overtones, the timbre of musical sounds, and the spectral properties of speech vowels (Luo and Poeppel, 2007; Giraud and Poeppel, 2012). Furthermore, theta-band dynamics contribute to the temporal organization of longer-lasting events, such as the melodic separation of auditory streams (Tóth et al., 2016; Kocsis, 2017). Consistent with the hemispheric specialization described by Flor-Henry et al. (2017), we interpret the right-hemispheric theta activity as an index of parallel spectral encoding, particularly of timbral and resonant characteristics. The observed right-lateralized encoding appears to reflect a continuous, fine-grained spectral mapping of acoustic qualities (Zatorre and Belin, 2001; Zatorre and Gandour, 2008; Hyde et al., 2008), distinct from the more abstract and global operations involved in auditory Gestalt formation. The more left-lateralized gamma activity, though not as pronounced as the right-lateralization of the theta activity, may reflect integrative operations of fast spectro-temporal binding and abstraction—functions relevant for auditory Gestalt formation in both non-verbal domains (e.g., fundamental pitch perception) and speech-related contexts (e.g., identification of temporally fluctuating phonemes).

Within this framework, the pronounced right-hemispheric theta activity observed in individuals with a marked preference for overtone listening may be viewed as a neural correlate of immediate high-resolution spectral encoding. The capacity for overtone listening and singing could thus be interpreted as an enhanced right-lateralized auditory mode, emphasizing the persistence of fine-grained harmonic structural details and separate auditory streams (Tóth et al., 2016; Kocsis, 2017) over fast spectro-temporal Gestalt integration (Luo and Poeppel, 2007; Giraud and Poeppel, 2012; Poeppel and Assaneo, 2020). This lateralization pattern supports the view that theta and gamma oscillations subserve distinct yet complementary functions in auditory perception and cognition, possibly reflecting a dual mode of representation: one based on spectral details and internal resonance, the other on dynamic grouping and spectro-temporal abstraction.

The present findings support the notion that auditory processing may rely on two complementary modes of neural representation. Right-hemispheric theta activity, particularly prominent in individuals with a preference for overtone listening, may reflect a detail-preserving, resonance-based encoding of spectral information. In contrast, left-hemispheric gamma activity may be associated with temporally structured integration and the abstraction of auditory objects. This distinction goes beyond the classical dichotomy of speech-related left-hemispheric and music-related right-hemispheric processing and suggests a more fundamental organizational principle: one mode emphasizes immediate, continuous mapping of acoustic features, the other operates through fast temporal integration and categorical abstraction (Zatorre et al., 2002). Both modes may serve distinct yet interdependent functions in auditory perception and cognition.

### Individual pitch perception style shapes stimulus-driven neural dynamics

In addition to state-dependent dynamics, our results highlight the role of trait-like auditory dispositions. The PPPT index—a measure of individual preference for overtone versus fundamental pitch perception—emerged as a strong predictor of both oscillatory power and hemispheric lateralization. Listeners with a spectral (overtone-focused) perceptual style showed stronger theta-band power and more pronounced right-hemispheric dominance across all studied conditions. Crucially, these individual dispositions modulated the pattern of stimulus-driven neural effects. Spectral listeners not only showed an elevated theta-band activity in response to the presented stimuli, in general, but also a steeper increase in theta power and lateralization with growing overtone salience. This supports a dynamic model in which stable perceptual styles influence the brain’s responsiveness to acoustic changes, and thus can be interpreted as an indicator of perceptual flexibility.

These findings extend earlier MEG research showing enhanced gamma-band responses in fundamental pitch listeners during tasks involving ambiguous pitch-timbre relations (Schulte et al., 2002). Other studies have reported gamma enhancement for consonant in contrast to dissonant musical intervals, suggesting that harmonic salience may engage distinct frequency bands depending on perceptual style and musical context (e.g., Park et al., 2011; Passynkova et al., 2007). Notably, both fundamental pitch perception and the experience of musical consonance are grounded in the same acoustic principle—namely, the alignment of harmonic partials via greatest common denominators in the frequency spectrum, which facilitates neuronal synchronization (Seither-Preisler et al., 2003, 2006a), particularly in the gamma-band range (Schulte et al., 2002).

Moreover, pitch perception modes have been linked to hemispheric asymmetries, instrumental preferences, and auditory expertise (Schneider et al., 2005a, 2005b; Seither-Preisler et al., 2007). The present data unify these strands by showing that overtone salience, neural oscillations, and perceptual mode are tightly interlinked. Importantly, the findings validate the resonance-based vowel model on a neurophysiological level—showing that vowel formants, when perceived as spectral pitches, engage dynamic and individualized patterns of cortical activity. It should be acknowledged that the modest sample size (n = 17) and the specific focus on overtone singers may limit the generalizability of our findings beyond this specialized cohort, including to other types of trained musicians. Accordingly, our results should be regarded as exploratory and hypothesis-generating, providing a foundation for future studies with larger and more diverse samples.

### Practical implications and future directions

The present findings offer new perspectives for applied neuroscience, vocal pedagogy, and sound-based interventions. Our findings demonstrate that overtone-rich stimuli can rapidly modulate oscillatory brain states even in the absence of active tasks, with a transition from gamma- to theta-dominant processing occurring within seconds. This reflects an accessible, non-invasive pathway to shifting perceptual focus, enhancing right-hemispheric integration, and capturing the neurodynamic flexibility of the auditory system in response to subtle spectral shifts.

Theta and gamma oscillations are implicated in a range of auditory and cognitive operations—including attention, memory, and prediction. Theta-band oscillations have been consistently associated with internally directed attention and altered modes of awareness, including hypnotic absorption (Paoletti et al., 2020; Jensen et al., 2015), trance (Flor-Henry et al., 2017), meditative states (Lomas et al., 2015), emotional regulation, cognitive flexibility, and access to inner imagery (Lomas et al., 2015; Jensen et al., 2015). Flor-Henry et al. (2017) reported increased theta-band activity in the right hemisphere during shamanic trance induced by rhythmic musical stimulation, a pattern aligning with the right-lateralized theta activity observed in our study during overtone-focused listening.

These findings suggest that overtone-rich auditory stimulation may offer an additional, low-threshold means of facilitating relaxed and internalized states of consciousness. This approach could serve as a further option for inducing beneficial brain states, potentially useful in psychotherapeutic, clinical, and educational settings, and contributing to enhanced self-awareness, stress reduction, creativity enhancement, and overall well-being. Future studies are encouraged to investigate these effects in specific non-clinical and clinical target groups.

In the context of vocal pedagogy, the pitch-based vowel diagram provides a concrete method to optimize vocal tract configurations for resonance alignment. Unlike conventional vowel descriptions based on perceptual or articulatory phonetics, this approach is grounded in pitch perception of resonances and enables precise control over vocal timbre and intonation—particularly valuable for high-level professional singing, ensemble work and overtone singing. Moreover, the strong association between the PPPT index and neural responsiveness suggests that individualized auditory training protocols could be developed. These may include targeted auditory exercises or neurofeedback approaches to enhance resonance-based auditory awareness and voice control competence, as well as theta-based perceptual and cognitive functions. Overtone listening or vocal training may thus serve as effective components within a multimodal approach to promote beneficial brain states relevant for both therapeutic and educational purposes. Future research should explore whether sustained overtone training induces long-term structural and/or functional plasticity in auditory cortex, similar to effects observed in instrumental musicians (Schlaug et al., 1995; Schneider et al., 2002, 2023; Gaser and Schlaug, 2003; Hyde et al., 2009; Groussard et al., 2010; Vaquero et al., 2016; Giacosa et al., 2016). Longitudinal studies could assess the impact of regular overtone practice on attention, emotion, and learning outcomes across clinical and non-clinical populations.

## Conclusion

This study provides converging neurophysiological and acoustic evidence that vowel resonance perception is not a fixed capacity but a flexible auditory skill, grounded in the interplay between spectral cues and neural oscillations. A resonance-based auditory paradigm induced a clear shift in cortical processing from gamma- to theta-dominant activity, accompanied by increasing right-hemispheric lateralization. These dynamics were modulated both by stimulus structure and by individual perceptual profiles for musical sounds and complex tones, as indexed by the PPPT.

Beyond the classical verbal–musical dichotomy, the present findings may point to a more fundamental organizational principle in auditory perception and cognition. While the left hemisphere appears to engage in segmentation, abstraction, and goal-directed processing, the right hemisphere may support a resonance-based, parallel mode of perception that facilitates fine-grained alignment with acoustic reality. These complementary modes could reflect two sides of auditory perception and cognitive functioning: one that abstracts and acts, and one that listens and resonates.

## Data Availability

The raw data supporting the conclusions of this article will be made available by the authors, without undue reservation.

## Glossary

Harmonic Vowels: Vowels where the resonance frequencies (*f*_R1_ and *f*_R2_) align with specific partial frequencies of the singing voice, sharing integer harmonic ratios.
Non-Harmonic Vowels: Vowels where one or both resonance frequencies do not align with partials, resulting in a more dampened sound.
Partials: Individual frequency components of a complex sound, including the fundamental and overtones.
Harmonics: Partial with integer frequency ratios.
Formants: Frequencies at peaks of an envelope curve around an amplitude distribution of the partials of a sound caused by resonances.
Vocal Tract: The anatomical area involved in sound production, including the mouth, nose, pharynx, and larynx.
Articulator: Parts of the vocal tract that are responsible for articulation of speech sounds.
Resonance Frequency: The frequency at which a system naturally vibrates, maximising energy transfer.
Acoustic-Phonetic Vowel Triangle: A diagram showing the relationship between the first two formant frequencies of different vowels.
